# A Deep Learning Approach Validates Genetic Risk Factors for Late Toxicity After Prostate Cancer Radiotherapy in a REQUITE Multi-National Cohort

**DOI:** 10.3389/fonc.2020.541281

**Published:** 2020-10-15

**Authors:** Michela Carlotta Massi, Francesca Gasperoni, Francesca Ieva, Anna Maria Paganoni, Paolo Zunino, Andrea Manzoni, Nicola Rares Franco, Liv Veldeman, Piet Ost, Valérie Fonteyne, Christopher J. Talbot, Tim Rattay, Adam Webb, Paul R. Symonds, Kerstie Johnson, Maarten Lambrecht, Karin Haustermans, Gert De Meerleer, Dirk de Ruysscher, Ben Vanneste, Evert Van Limbergen, Ananya Choudhury, Rebecca M. Elliott, Elena Sperk, Carsten Herskind, Marlon R. Veldwijk, Barbara Avuzzi, Tommaso Giandini, Riccardo Valdagni, Alessandro Cicchetti, David Azria, Marie-Pierre Farcy Jacquet, Barry S. Rosenstein, Richard G. Stock, Kayla Collado, Ana Vega, Miguel Elías Aguado-Barrera, Patricia Calvo, Alison M. Dunning, Laura Fachal, Sarah L. Kerns, Debbie Payne, Jenny Chang-Claude, Petra Seibold, Catharine M. L. West, Tiziana Rancati

**Affiliations:** ^1^Modelling and Scientific Computing Laboratory, Math Department, Politecnico di Milano, Milan, Italy; ^2^Center for Analysis, Decisions and Society, Human Technopole, Milan, Italy; ^3^Medical Research Council-Biostatistic Unit, University of Cambridge, Cambridge, United Kingdom; ^4^CHRP-National Center for Healthcare Research and Pharmacoepidemiology, University of Milano-Bicocca, Milan, Italy; ^5^Department of Human Structure and Repair, Ghent University, Ghent, Belgium; ^6^Department of Radiation Oncology, Ghent University Hospital, Ghent, Belgium; ^7^Leicester Cancer Research Centre, Department of Genetics and Genome Biology, University of Leicester, Leicester, United Kingdom; ^8^Department of Radiation Oncology, University Hospitals Leuven, Leuven, Belgium; ^9^Maastricht University Medical Center, Maastricht, Netherlands; ^10^Department of Radiation Oncology (Maastro), GROW Institute for Oncology and Developmental Biology, Maastricht, Netherlands; ^11^Translational Radiobiology Group, Division of Cancer Sciences, Manchester Academic Health Science Centre, Christie Hospital, University of Manchester, Manchester, United Kingdom; ^12^Department of Radiation Oncology, Universitätsmedizin Mannheim, Medical Faculty Mannheim, Heidelberg University, Mannheim, Germany; ^13^Department of Radiation Oncology 1, Fondazione IRCCS Istituto Nazionale dei Tumori, Milan, Italy; ^14^Department of Medical Physics, Fondazione IRCCS Istituto Nazionale dei Tumori, Milan, Italy; ^15^Department of Oncology and Haemato-Oncology, University of Milan, Milan, Italy; ^16^Prostate Cancer Program, Fondazione IRCCS Istituto Nazionale dei Tumori, Milan, Italy; ^17^Department of Radiation Oncology, University Federation of Radiation Oncology, Montpellier Cancer Institute, Univ Montpellier MUSE, Grant INCa_Inserm_DGOS_12553, Inserm U1194, Montpellier, France; ^18^Department of Radiation Oncology, University Federation of Radiation Oncology, CHU Caremeau, Nîmes, France; ^19^Department of Radiation Oncology, Icahn School of Medicine at Mount Sinai, New York, NY, United States; ^20^Department of Genetics and Genomic Sciences, Icahn School of Medicine at Mount Sinai, New York, NY, United States; ^21^Fundación Pública Galega de Medicina Xenómica, Grupo de Medicina Xenómica (USC), Santiago de Compostela, Spain; ^22^Instituto de Investigación Sanitaria de Santiago de Compostela, Santiago de Compostela, Spain; ^23^Biomedical Network on Rare Diseases (CIBERER), Madrid, Spain; ^24^Department of Radiation Oncology, Complexo Hospitalario Universitario de Santiago, SERGAS, Santiago de Compostela, Spain; ^25^Strangeways Research Labs, Department of Oncology, Centre for Cancer Genetic Epidemiology, University of Cambridge, Cambridge, United Kingdom; ^26^Wellcome Sanger Institute, Wellcome Genome Campus, Hinxton, United Kingdom; ^27^Departments of Radiation Oncology and Surgery, University of Rochester Medical Center, Rochester, New York, NY, United States; ^28^Centre for Integrated Genomic Medical Research (CIGMR), University of Manchester, Manchester, United Kingdom; ^29^Division of Cancer Epidemiology, German Cancer Research Center (DKFZ), Heidelberg, Germany; ^30^University Cancer Center Hamburg, University Medical Center Hamburg-Eppendorf, Hamburg, Germany

**Keywords:** prostate cancer, late toxicity, snps, deep learning, autoencoder, validation

## Abstract

**Background:** REQUITE (validating pREdictive models and biomarkers of radiotherapy toxicity to reduce side effects and improve QUalITy of lifE in cancer survivors) is an international prospective cohort study. The purpose of this project was to analyse a cohort of patients recruited into REQUITE using a deep learning algorithm to identify patient-specific features associated with the development of toxicity, and test the approach by attempting to validate previously published genetic risk factors.

**Methods:** The study involved REQUITE prostate cancer patients treated with external beam radiotherapy who had complete 2-year follow-up. We used five separate late toxicity endpoints: ≥grade 1 late rectal bleeding, ≥grade 2 urinary frequency, ≥grade 1 haematuria, ≥ grade 2 nocturia, ≥ grade 1 decreased urinary stream. Forty-three single nucleotide polymorphisms (SNPs) already reported in the literature to be associated with the toxicity endpoints were included in the analysis. No SNP had been studied before in the REQUITE cohort. Deep Sparse AutoEncoders (DSAE) were trained to recognize features (SNPs) identifying patients with no toxicity and tested on a different independent mixed population including patients without and with toxicity.

**Results:** One thousand, four hundred and one patients were included, and toxicity rates were: rectal bleeding 11.7%, urinary frequency 4%, haematuria 5.5%, nocturia 7.8%, decreased urinary stream 17.1%. Twenty-four of the 43 SNPs that were associated with the toxicity endpoints were validated as identifying patients with toxicity. Twenty of the 24 SNPs were associated with the same toxicity endpoint as reported in the literature: 9 SNPs for urinary symptoms and 11 SNPs for overall toxicity. The other 4 SNPs were associated with a different endpoint.

**Conclusion:** Deep learning algorithms can validate SNPs associated with toxicity after radiotherapy for prostate cancer. The method should be studied further to identify polygenic SNP risk signatures for radiotherapy toxicity. The signatures could then be included in integrated normal tissue complication probability models and tested for their ability to personalize radiotherapy treatment planning.

## Introduction

Radiotherapy represents the most effective non-surgical modality for the potentially curative treatment of prostate cancer. Around a half of survivors underwent radiotherapy as part of their curative care (1), either as single curative treatment or as adjuvant/salvage treatment after radical prostatectomy.

Despite the fact that prognosis is very good in terms of patients' survival rates, it is widely acknowledged that long-term side-effects after radiotherapy can affect a patient's quality-of-life (2–4). A tool able to identify patients likely to develop toxicity could be a crucial step toward personalized radiotherapy with modification of the dose, fractionation, techniques and supportive care. The ultimate goal is to reduce morbidity and improve quality-of-life.

Radiation toxicity is a multifactorial problem, related not only to the cumulative delivered dose, but also to an intrinsic process within tissues responding to cellular injury. Individual genetic background and biological expression pattern, premorbid conditions, concomitant oncological therapies, as well as the cellular microenvironment, could be important factors in the development of side-effects, although their exact contributions are unknown.

With increased interest in this field and relevant data collection on this topic, predictive models have been developed to identify patients likely to develop side effects during radiotherapy (3).

The identification of genetic factors associated with susceptibility to radiation toxicity represents an emerging research area in oncology. A number of different approaches have been explored (5–13), however, the developed models and biomarkers have failed to progress to routine clinical use due to the lack of thorough independent validation.

REQUITE (validating pREdictive models and biomarkers of radiotherapy toxicity to reduce side effects and improve QUalITy of lifE in cancer survivors) was established with the aim of validating models and biomarkers for the prediction of adverse effects following radiotherapy (14–16). In order to address previous limitations in pooling data, in using common toxicity scoring systems and in collecting standardized data, REQUITE carried out an international, multi-center, prospective observational study. A centralized biobank was also established to store blood samples and genome-wide genotyping of single nucleotide polymorphisms (SNPs) was carried out.

The specific purpose of the present study was to attempt to validate genetic risk factors for late toxicity (rectal bleeding and late urinary symptoms) after prostate cancer radiotherapy in the REQUITE population using a deep learning algorithm. This technique aims to identify patient-specific features that define patients with toxicity (“unhealthy”) as outliers with respect to the population of irradiated patients without toxicity (“healthy”).

Deep learning has the potential to overcome the difficulties in replication of results faced by the widespread single-SNP association methods used by genome wide association studies (GWAS). The statistical power of GWAS is limited by a combination of the large number of hypotheses being tested simultaneously and the inherently small effect size of the single SNP (17).

Deep learning approaches, with their intrinsic hierarchical structure (where each layer performs a combination of the outcomes of the previous layers), seem particularly adapt at mimicking complex dependencies within data. The method addresses effectively the following issues: (i) unstable selections of correlated variables and inconsistent selections of linearly dependent genetic variables (18); (ii) strong imbalance between positive and negative outcomes which is usually encountered in studies of radiation toxicity.

## Materials and Methods

### Population

REQUITE prostate cancer patients treated with external beam radiotherapy (with/without hormonal therapy, with/without a previous prostatectomy, no brachytherapy) and complete 2-year follow-up were included. Details on the REQUITE population are given in Seibold et al. (14).

Prostate cancer patients were recruited prior to radiotherapy between April 2014 and October 2016. Recruitment was at ten main sites in eight countries (Belgium, France, Germany, Italy, the Netherlands, Spain, UK, US). Conventionally fractionated or hypo-fractionated radiotherapy was prescribed according to local standard-of-care regimens. The patients were followed prospectively for at least 24 months, with longer follow-up encouraged where possible. All patients gave written informed consent. The study was approved by local Ethical Committees and is registered at www.controlled-trials.com (ID ISRCTN98496463).

Demographic, co-morbidity, treatment, physics, longitudinal toxicity (CTCAE v4.0 healthcare professional and patient reported), quality-of-life, and treatment outcome data were collected prospectively using standardized case report forms. CTCAE v4.0 based questionnaires developed to collect patient reported outcomes were adapted from those published elsewhere for the male pelvis (19) and updated to fit with CTCAE v4.0 items.

All patients donated at least two blood samples prior to the start of radiotherapy: an EDTA sample for SNP genotyping plus a PAXgene sample. Genotyping data were generated using the Illumina Infinium OncoArray-500K beadchip. Following standard quality control procedures (20), genotype data were imputed using the 1,000 Genomes Project (version 3) as a reference panel.

### Selection of Genetic Risk Factors

We undertook a comprehensive search of Medline and PubMed databases using the keywords “prostate,” “prostatic,” “radiotherapy,” “radiation,” “irradiation,” “toxicity,” “adverse effects,” “side-effects,” “morbidity,” “injury,” “genetic variation,” “SNP,” “GWAS,” and “polymorphism.” This search identified 60 SNPs published (up to May 31st, 2019) in GWAS patient studies with *p* < 1.0·10^−5^ and where findings were adjusted for multiple comparisons OR in studies including a controlled number of SNPs (~10^2^) and using multivariable regularization methods coupled to internal validation to control overfitting.

Forty-three of 60 SNPs were available for the REQUITE population (either directly determined or after imputation) and were included in the analysis. These SNPs were identified in five papers (5, 11, 21–23) and the full list is reported in Table 1.

**Table 1:** Full list of SNPs selected from the literature for validation and associated toxicity endpoint following prostate radiotherapy.

**SNP**	**OR**	***p*-value**	**References**
**Rectal bleeding**			
rs10519410	3.7	1.3 × 10^−6^	(21)
rs17055178	1.95^#^	6.2 × 10^−10^	(23)
**Urinary frequency**			
rs17599026	3.12	4.16 × 10^−8^	(5)
rs342442	0.51	3.86 × 10^−7^	(5)
rs8098701	2.41	2.11 × 10^−6^	(5)
rs7366282	3.2	2.03 × 10^−6^	(5)
rs10209697	2.66	2.27 × 10^−6^	(5)
rs4997823	0.49	2.35 × 10^−6^	(5)
rs7356945	1.74	3.71 × 10^−6^	(5)
rs6003982	0.51	4.28 × 10^−6^	(5)
rs10101158	1.8	4.39 × 10^−6^	(5)
**Decreased urinary stream**			
rs7720298	2.71	3.21 × 10^−8^	(5)
rs17362923	2.7	6.79 × 10^−7^	(5)
rs76273496	3.68	2.71 × 10^−6^	(5)
rs144596911	3.6	2.94 × 10^−6^	(5)
rs62091368	4.36	3.95 × 10^−6^	(5)
rs141342719	3.5	3.97 × 10^−6^	(5)
rs673783	2.49	4.33 × 10^−6^	(5)
rs10969913	3.92^#^	2.9 × 10^−10^	(23)
**Haematuria**			
rs11122573	1.92^#^	1.8 × 10^−8^	(23)
rs708498	0.24	n.a.^§^	(22)
rs845552	0.95	n.a.^§^	(22)
**Nocturia**			
rs1799983	0.19	n.a.^§^	(22)
rs1045485	0.27	n.a.^§^	(22)
**Overall toxicity (STAT^#^ score)**			
rs10497203^*^	1.48	8.84 × 10^−11^	(11)
rs7582141^*^	1.45	4.64 × 10^−11^	(11)
rs6432512^*^	1.42	1.97 × 10^−10^	(11)
rs264651^*^	1.49	1.48 × 10^−7^	(11)
rs264588^*^	1.45	3.08 × 10^−10^	(11)
rs264631^*^	1.43	6.4 × 10^−10^	(11)
rs147596965	1.95	6.19 × 10^−8^	(5)
rs77530448	1.43	7.36 × 10^−8^	(5)
rs4906759	1.73	1.55 × 10^−7^	(5)
rs71610881	1.82	5.41 × 10^−7^	(5)
rs141799618	1.55	1.22 × 10^−6^	(5)
rs2842169	1.32	1.45 × 10^−6^	(5)
rs11219068	1.32	1.74 × 10^−6^	(5)
rs8075565	1.32	2.20 × 10^−6^	(5)
rs6535028	1.34	2.70 × 10^−6^	(5)
rs4775602	1.26	3.20 × 10^−6^	(5)
rs7829759	1.39	3.84 × 10^−6^	(5)
rs79604958	1.60	4.33 × 10^−6^	(5)
rs12591436	1.20	5.66 × 10^−6^	(5)

# *overall toxicity as defined by calculating the Standardized Total Average Toxicity (STAT) score (24)*.

* *All these variants are highly correlated in European populations and represent the same association signal. See also correlation matrix as determined in the REQUITE population in the Supplementary Figure 1*.

# *Hazard Ratio*.

§ *SNPs were selected using Least Absolute Shrinkage and Selection Operator (LASSO) multivariable regression out of a panel of 384 previous identified SNPs, p-value not available*.

### Outcome Endpoints

Toxicity endpoints were defined using CTCAE v4.0 scoring reported by health professionals or Patient Reported Outcomes, as detailed for each single endpoint. As the frame of the DSAE is to identify SNPs who would tag a patient as exceptionally “sensitive” to radiation (an “outlier”), patients with other possible known intrinsic higher risk of exhibiting radiation toxicity were always excluded, in particularly patients who had systemic lupus erythematosus, rheumatoid arthritis and other collagen vascular diseases.

The following endpoints were considered:

1. Late rectal bleeding grade≥1 (CTCAE v4.0 scoring): patients exhibiting at least mild bleeding (even requiring no intervention) at 12 or at 24 months. Patients with grade≥1 at baseline and grade ≤ 1 during follow-up were considered as not bleeders; patients with hemorrhoids before radiotherapy treatment were excluded.
2. Late urinary frequency grade≥2 (CTCAE v4.0 scoring): patients with urinary frequency limiting instrumental activities of daily living or if urinary frequency requiting medical management at 12 or at 24 months. Patients with urinary frequency grade≥2 at baseline and grade ≤ 2 during follow-up were considered as not exhibiting this endpoint.
3. Late haematuria grade ≥1 (CTCAE scoring): patients with asymptomatic haematuria (clinical or diagnostic observations only, no intervention indicated) at 12 or 24 months. Patients with haematuria grade≥1 at baseline and grade ≤ 1 during follow-up were considered as not exhibiting the endpoint.
4. Late nocturia grade ≥2 (Patient Reported Outcome): patients declaring need to urinate at least two-three times per night at 12 or 24 months. Patients with nocturia grade≥2 at baseline and grade ≤ 2 during follow-up were considered as not exhibiting the endpoint.
5. Late grade≥1 (Patient Reported Outcome): patients scored with hesitant or dripping stream at 12 or 24 months. Patients with decreased urinary stream grade≥1 at baseline and grade ≤ 1 during follow-up were considered as not exhibiting the endpoint.

Patients who underwent transurethral resection of the bladder and patients on anti-muscarinic drugs (factors which could constitute a confounding factor in the scoring of urinary toxicity) were excluded when considering all urinary endpoints.

### Deep Sparse AutoEncoder for SNPs Validation

The methodology described in Massi et al. (25) was considered. This method proposes a novel feature selection algorithm for the minority class in an imbalanced dataset, i.e., in cases like this dataset, where there is a strong imbalance between the number of patients that are scored as healthy (without side effects) vs. unhealthy (with side effects). The approach uses a representation learning technique, specifically a Deep Sparse AutoEncoder, to obtain the best representation of the majority class (healthy patients in this dataset) and to consequently identify which features (SNPs) distinguish the minority class (unhealthy patients) with respect to the majority class.

An AutoEncoder (AE) is a neural network with an output that reconstructs the input (26). In its simplest version an AE is composed of the input, the output and only a single hidden layer. The input layer in our case is composed of *J* nodes, one per feature (one per SNP), and we consider a data matrix *X*, in which each row **x**_*i*_ is the vector of SNPs recorded for the patient *i, i* ∈ {1 *,., N*}. The input layer is connected to the hidden layer, **h**_*i*_, through the *encoder* function, *f*, such that **h**_*i*_ = *f* (**Wx***_i_* +**b**); here **W** ∈ R ^*H* × *J*^ denotes the weight matrix and **b** ∈ R^*H* × 1^ the bias vector. Then, the output is the result of the application of a *decoder* function, *g*, to the hidden layer **h**_*i*_, such that $\hat{\text{x}}$_*i*_ = *g* (**W'h***_i_* +**b'**), where **W'** ∈ R ^*J* × *H*^ is the weight matrix and **b** ∈ R ^*J* × 1^ is the bias vector. Having fixed the functions *f* and *g*, the training of the network consists in estimating the corresponding optimal parameters (**W**, **b**, **W'**, **b'**), by minimizing the loss function *L*(**x**_*i*_, $\hat{\text{x}}$_*i*_), which is a function that gives a measure of the similarity between the input and the reconstructed output. In this work, we considered the Euclidean distance as loss function *L*.

**Figure 1:**
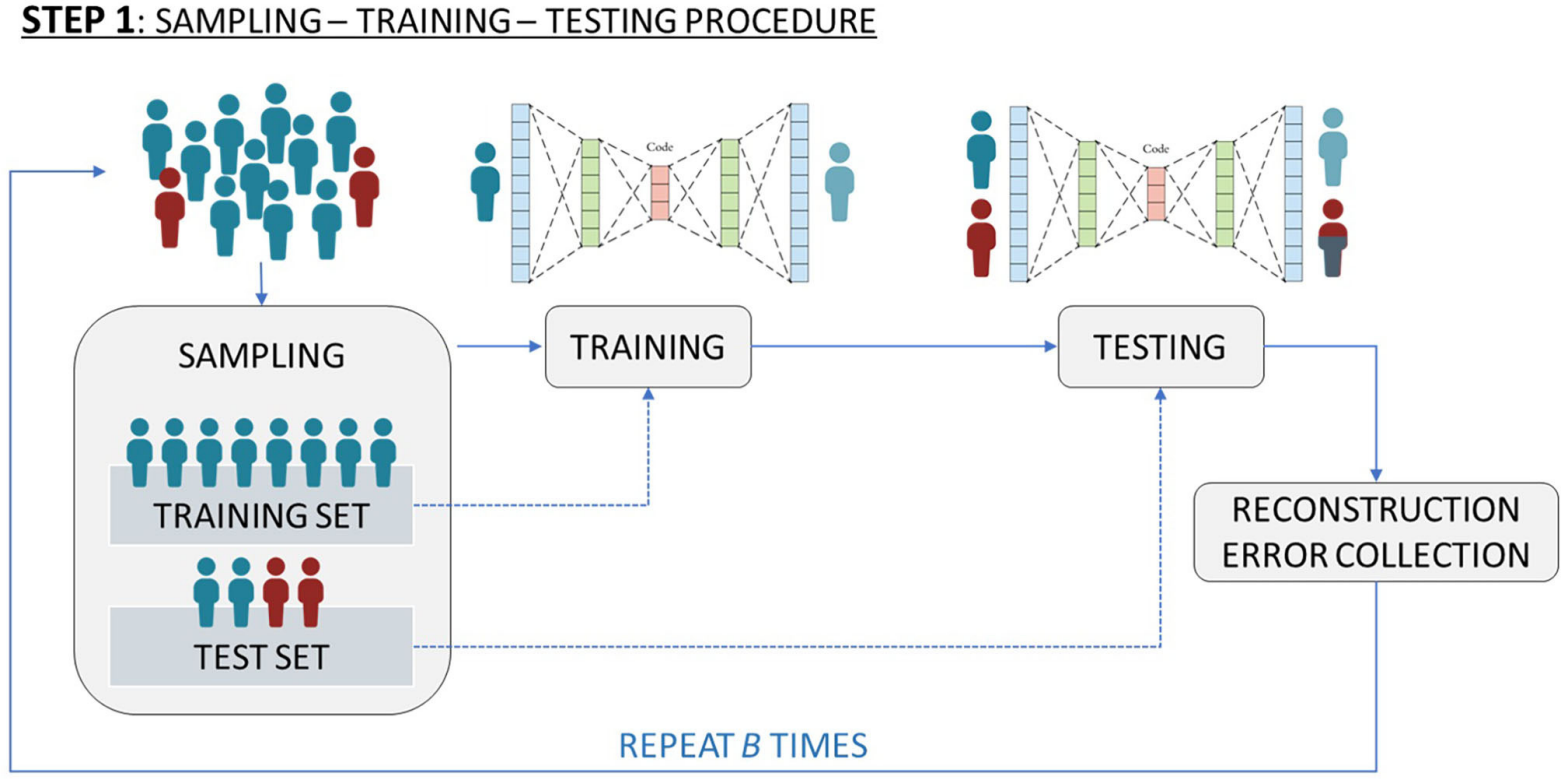
Simplified scheme of a Deep AutoEncoder.

A more sophisticated version of AE (named Deep AE) has *multiple* hidden layers in which the output of a layer is the input of the next one. Figure 1 depicts a simplified scheme of a Deep AutoEncoder.

In order to get an *effective reconstruction* of the input, that allows selection of features that best characterize the input data, we included a penalization term in the loss function. AE algorithms of this type are known as Deep Sparse AEs. Given this framework and with the final goal of validating the SNPs effect on the long-term radiation toxicity, we applied the previously described Deep Sparse AE as follows:

(i) *sampling*: we sampled *S healthy* patients (those without toxicity) where *S* equals the total number of *unhealthy* patients (those with toxicity). All the unhealthy patients and the *S* sampled healthy patients form the *test set*. All the remaining healthy patients constitute the *training set*.
(ii) *training*: we trained the network only on the previously specified training set. The idea here was to *learn* how to best represent healthy patients. The result of this step is the estimate of the neural network characteristics (weight and bias vectors, encoder and decoder functions).
(iii) *testing*: we tested the estimated network on the previously specified test set. The result of this step is a matrix of Reconstruction Errors, *R* ∈ R^(2S) × *J*^. Considering the previous step and the fact that unhealthy patients are the minority class, the rows of R which are related to unhealthy patients should contain higher values with respect to those rows of *R* associated to healthy patients.
(iv) *SNP identification*: we identified which SNPs are associated with the *highest* Reconstruction Error. Further details on this step are given at the end of this section.

The steps (i)-(iii) are repeated 50 times in order to reduce a possible selection bias induced by the sampling step (i), thus obtaining 50 *R* matrices.

**Figure 2:**
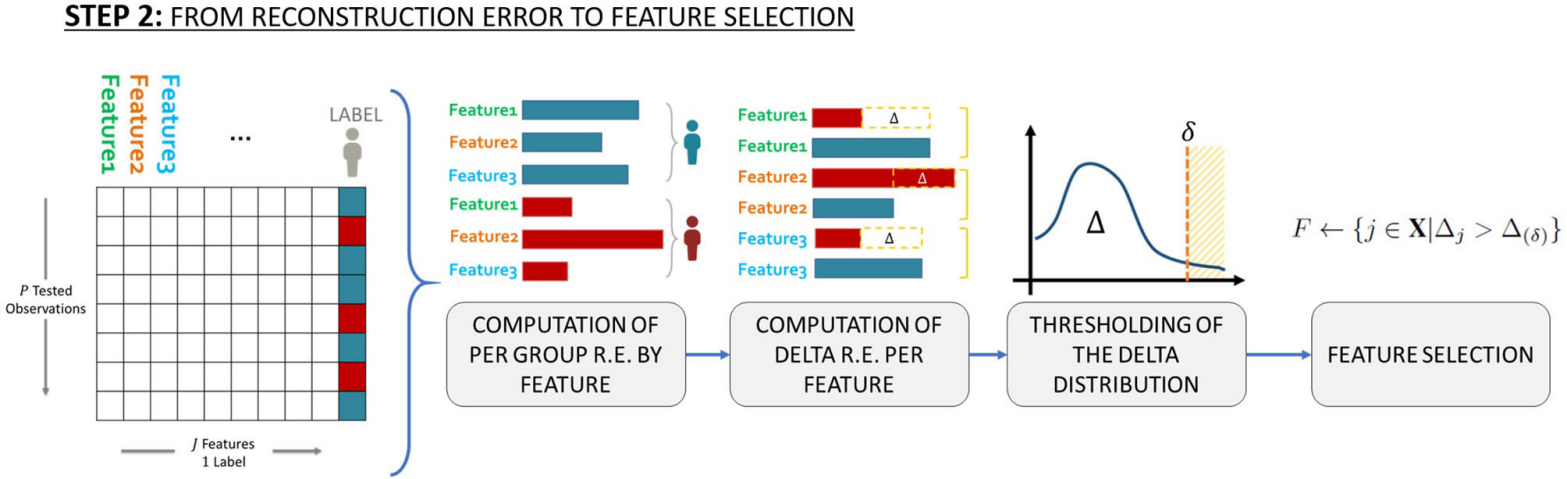
Schematic representation of the workflow used to identify which features to select to characterize the minority class (i.e., patients with toxicity) with respect to the majority class (patients without toxicity).

In order to identify which features should be selected for characterizing the minority class with respect to the majority class, in step (iv) the average Reconstruction Error per feature per class is computed according to that proposed in Massi et al. (25), which means computing two vectors (one for the unhealthy patients and one for the healthy patients), both made by *J* elements. Then, we investigated the distribution of the difference, Δ, between the average Reconstruction Errors related to unhealthy patients and the average Reconstruction Errors related to healthy patients. See Figure 2 for a schematic representation of the above described workflow.

Finally, to define which SNPs are associated with late toxicity endpoints, we set possible thresholds equal to the 70-th, 80-th, the 90-th and the 95-th percentiles of the distribution of the Reconstruction Error differences, Δ. This means that we investigated the SNPs associated with the top 30%, the top 20%, the top 10% and the top 5% differences. These thresholds identity the effect size of identified SNPs, a large effect size (Odds Ratio>2) for SNPs in the 90-th/95-th percentiles, a moderate (Odds Ratio~2) and small (Odds Ratio <2) effect size for SNPs in the 80-th and 70-th percentiles, respectively.

#### Architectural and Implementation Details

For the interested reader, in this section we provide some more specific details regarding the development and specific implementation of the DSAE for the applications described in this paper. For more details on the methodology, its strenghts and all model's hyperparameters mentioned below, refer to the description in Massi et al. (25).

The experiments were implemented and carried out using Python Keras framework for Deep Learning with Tensorflow as backend.

For better comparability of results in the experiments we structured the DSAEs included in the *sampling-training-testing* procedure with the same architecture and hyperparameters for all five endpoints. In particular, all the encoders of the DSAEs were composed of an input layer with *J* = *43* nodes (one per SNP), followed by a sequence of hidden layers of 40, 30 (with hyperbolic tangent activation function) and 20 nodes, respectively. To the 20 nodes of the innermost hidden layer we applied a sigmoidal activation function to foster the sparsity induced by the penalization term (weighted with λ=10e-5). The decoder architecture of all DSAEs was specular to the encoder, with a sequence of layers with 30 and 40 nodes, followed by an output layer of *J* = 43 nodes. The training of the DSAE for each of the B = 50 iterations was performed for 400 epochs, exploiting the Adam optimization algorithm with its default parameters (*learning rate* equal to 0.001).

## Results

### Cohort

REQUITE enrolled 1,681 prostate cancer patients who were treated with external beam radiotherapy without brachytherapy. One thousand four hundred and fifty patients with complete 2-year follow-up were available for analysis. Forty-nine patients were excluded because of an intrinsic higher risk of exhibiting radiation toxicity, due to their co-morbidities (patients with a diagnosis of systemic lupus erythematosus, rheumatoid arthritis and other collagen vascular diseases). Details on the clinical characteristics of the cohorts selected for each toxicity endpoint are given in Supplementary Tables 1,2.

### Validation of SNPs Associated With Late Toxicity Endpoints Through a Deep Sparse AutoEncoder

#### Late Rectal Bleeding grade≥1

**Figure 3:**
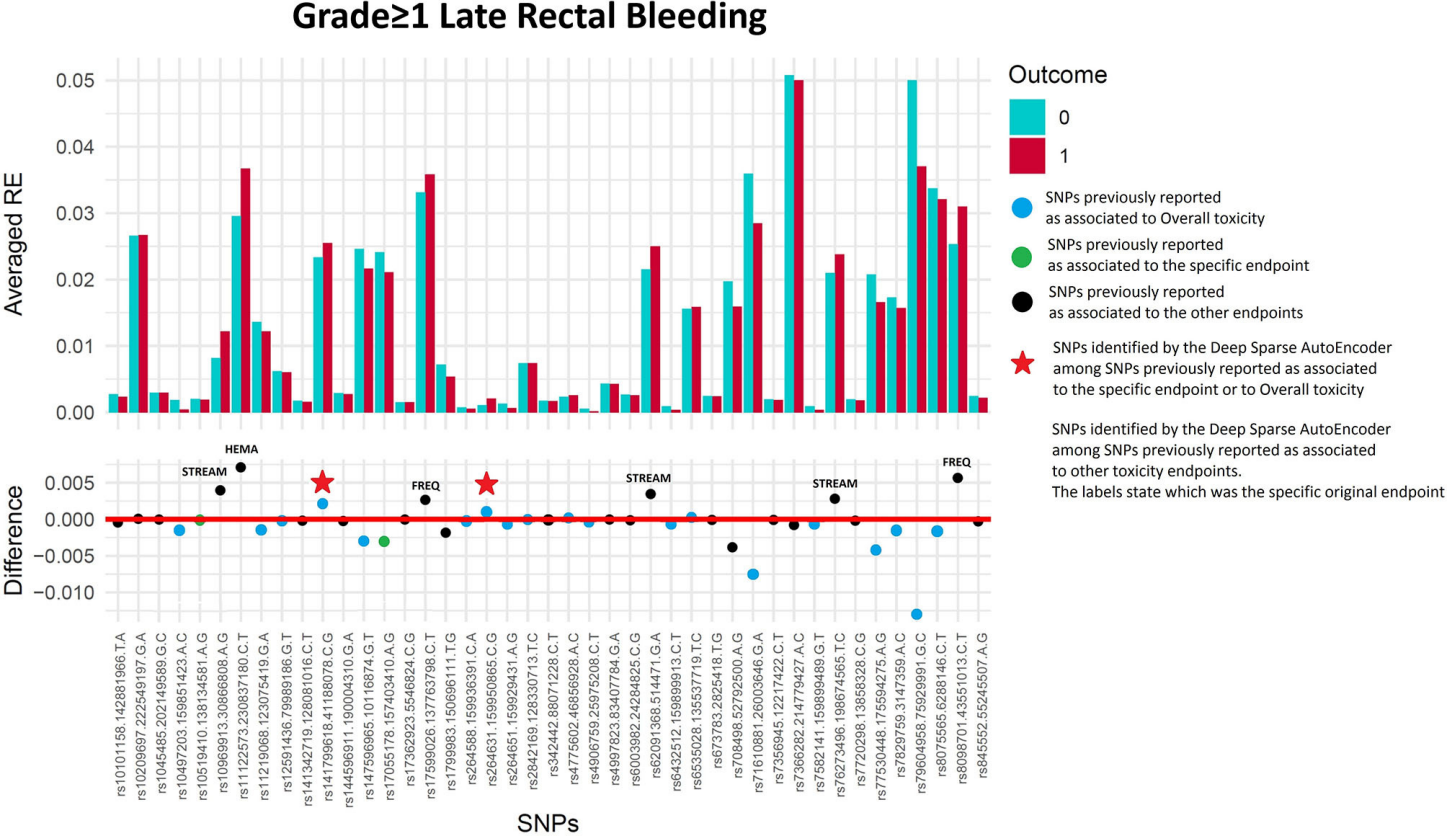
Results for late rectal bleeding grade≥1 from the Deep Sparse AutoEncoder. The 43 considered SNPs are reported in the x-axis and the averaged Reconstruction Errors (RE) are reported in the y-axis (top panel), red columns refer to patients with toxicity, while blue columns refer to patients without toxicity. In the lower panel the difference between averaged Reconstruction Errors between the two classes are represented for each SNP (i.e., differences between red and blue columns). For most SNPs, the difference is close to zero (red line in the bottom panel of the figure). The chosen thresholds in this difference (i.e., highest 30, 20, 10, and 5% differences) are selecting SNPs associated to the toxicity outcome. Green circles refer to SNPs that were previously identified as associated with late rectal bleeding, while blue circles refer to SNPs that were previously associated with overall toxicity as defined by calculation of the Standardized Total Average Toxicity (STAT) score (24). Red stars indicate SNPs (either specific for this endpoint or related to overall toxicity) defining patients with toxicity as outliers with respect to the characteristics of patients without toxicity. Labels show SNPs that not directly associated with late rectal bleeding/overall toxicity, but contributing to their identification. The label states for which toxicity endpoint the SNPs were originally associated with in the literature: FREQ=urinary frequency, HEMA=haematuria, NOCT=nocturia, STREAM=decreased urinary stream.

One hundred and sixty of 1,366 available patients (11.7%) had late rectal bleeding grade≥1. Figure 3 shows the differences between averaged Reconstruction Errors between the two classes (i.e., differences between red and blue columns). The largest part of the differences is close to zero (red line in the bottom panel of Figure 3). The chosen thresholds in this difference (i.e., highest 30, 20, 10, and 5% differences) select SNPs associated with the toxicity outcome with different effect size. Table 2 lists results for the SNPs previously reported to be associated with late rectal bleeding and overall toxicity in comparison with SNPs selected by the DSAE in the REQUITE cohort. For late rectal bleeding eight SNPs were identified, two SNPs previously associated with overall toxicity (red stars in Figure 3) and six SNPs previously found to be associated with urinary toxicity.

**Table 2:** Deep Sparse AutoEncoder testing of SNPs associated with Late Rectal Bleeding^*^.

**SNP**	**References**	**70-th percentile small effect size**	**80-th percentile moderate effect size**	**90-th percentile large effect size**	**95-th percentile large effect size**
**SNPs previously associated with late rectal bleeding**					
rs10519410	(21)	Not validated	Not validated	Not validated	Not validated
rs17055178	(23)	Not validated	Not validated	Not validated	Not validated
**SNPs previously associated with overall toxicity (STAT score)**					
rs264631	(11)	**Identified**	**Identified**	Not validated	Not validated
rs141799618	(5)	**Identified**	**Identified**	Not validated	Not validated

* *grade≥1 (all considered SNPs reported in the table) and to overall toxicity as defined by calculation of the Standardized Total Average Toxicity (STAT) score (24) (in this case only “Identified” SNPs were reported in the table). The SNPs that were correctly identified by the algorithm are flagged as “Identified”*.

#### Late Urinary Frequency Grade≥2

**Figure 4:**
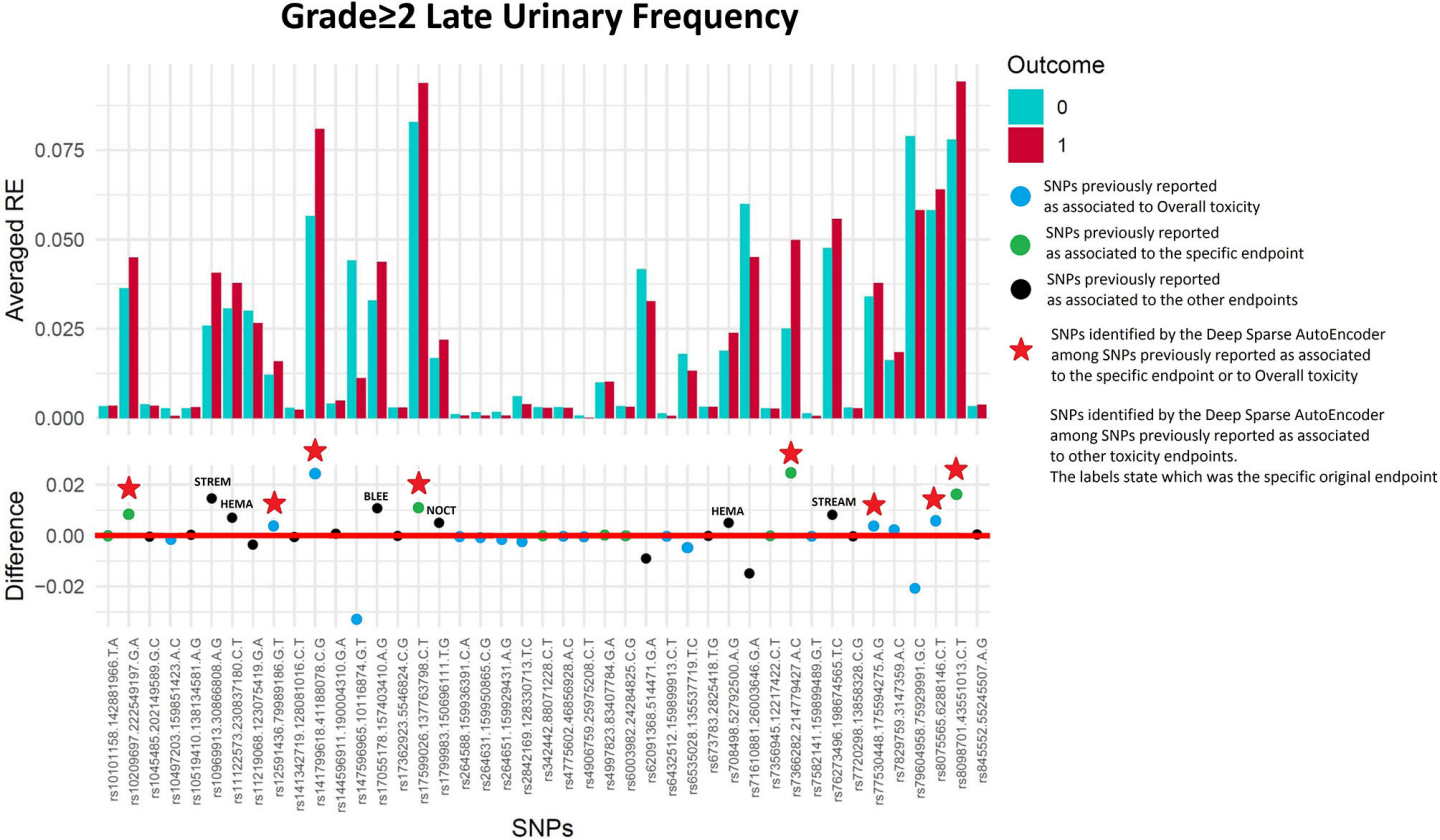
Results for late urinary frequency grade≥2 from the Deep Sparse AutoEncoder. The 43 considered SNPs are reported in the x-axis and the averaged Reconstruction Errors (RE) are reported in the y-axis (top panel), red columns refer to patients with toxicity, while blue columns refer to patients without toxicity. In the lower panel the difference between averaged Reconstruction Errors between the two classes are represented for each SNP (i.e., differences between red and blue columns). For most SNPs, the difference is close to zero (red line in the bottom panel of the figure). The chosen thresholds in this difference (i.e., highest 30, 20, 10, and 5% differences) are selecting SNPs associated to the toxicity outcome. Green circles refer to SNPs that were previously identified as associated with late urinary frequency, while blue circles refer to SNPs that were previously associated with overall toxicity as defined by calculation of the Standardized Total Average Toxicity (STAT) score (24). Red stars indicate SNPs (either specific for this endpoint or related to overall toxicity) defining patients with toxicity as outliers with respect to the characteristics of patients without toxicity. Labels show SNPs that not directly associated with late urinary frequency/overall toxicity, but contributing to their identification. The label states for which toxicity endpoint the SNPs were originally associated with in the literature: BLEE=rectal bleeding, HEMA=haematuria, NOCT=nocturia, STREAM=decreased urinary stream.

Fifty-six of 1,334 available patients (4.2%) experienced late urinary frequency grade≥2. Patients were excluded from the analysis if they had urinary frequency grade≥2 at baseline (*n* = 26), they underwent transurethral resection of the bladder (*n* = 31) or were using anti-muscarinic drugs (*n* = 10). Figure 4 and Table 3 show that the DSAE analysis identified 14 SNPs: four already reported as associated with urinary frequency (*rs17599026, rs8098701, rs7366282, rs10209697*), four associated with overall toxicity, one previously associated with bleeding and five with other urinary symptoms.

**Table 3:** Results from Deep Sparse AutoEncoder testing of SNPs associated with Urinary Frequency^*^.

**SNP**	**References**	**70-th percentile small effect size**	**80-th percentile moderate effect size**	**90-th percentile large effect size**	**95-th percentile large effect size**
**SNPs previously associated with late urinary frequency**					
rs17599026	(5)	**Identified**	**Identified**	**Identified**	Not validated
rs342442	(5)	Not validated	Not validated	Not validated	Not validated
rs8098701	(5)	**Identified**	**Identified**	**Identified**	**Identified**
rs7366282	(5)	**Identified**	**Identified**	**Identified**	**Identified**
rs10209697	(5)	**Identified**	**Identified**	Not validated	Not validated
rs4997823	(5)	Not validated	Not validated	Not validated	Not validated
rs7356945	(5)	Not validated	Not validated	Not validated	Not validated
rs6003982	(5)	Not validated	Not validated	Not validated	Not validated
rs10101158	(5)	Not validated	Not validated	Not validated	Not validated
**SNPs previously associated with overall toxicity (STAT score)**					
rs147596965	(5)	**Identified**	Not validated	Not validated	Not validated
rs77530448	(5)	**Identified**	**Identified**	**Identified**	**Identified**
rs8075565	(5)	**Identified**	Not validated	Not validated	Not validated
rs12591436	(5)	**Identified**	Not validated	Not validated	Not validated

* Late Urinary Frequency grade≥2 (all considered SNPs reported in the table) and to overall toxicity as defined by calculation of the Standardized Total Average Toxicity (STAT) score (24) (in this case only “Identified” SNPs were reported in the table). The SNPs that were correctly identified by the algorithm are flagged as “Identified.”

#### Late Haematuria Grade≥1

**Figure 5:**
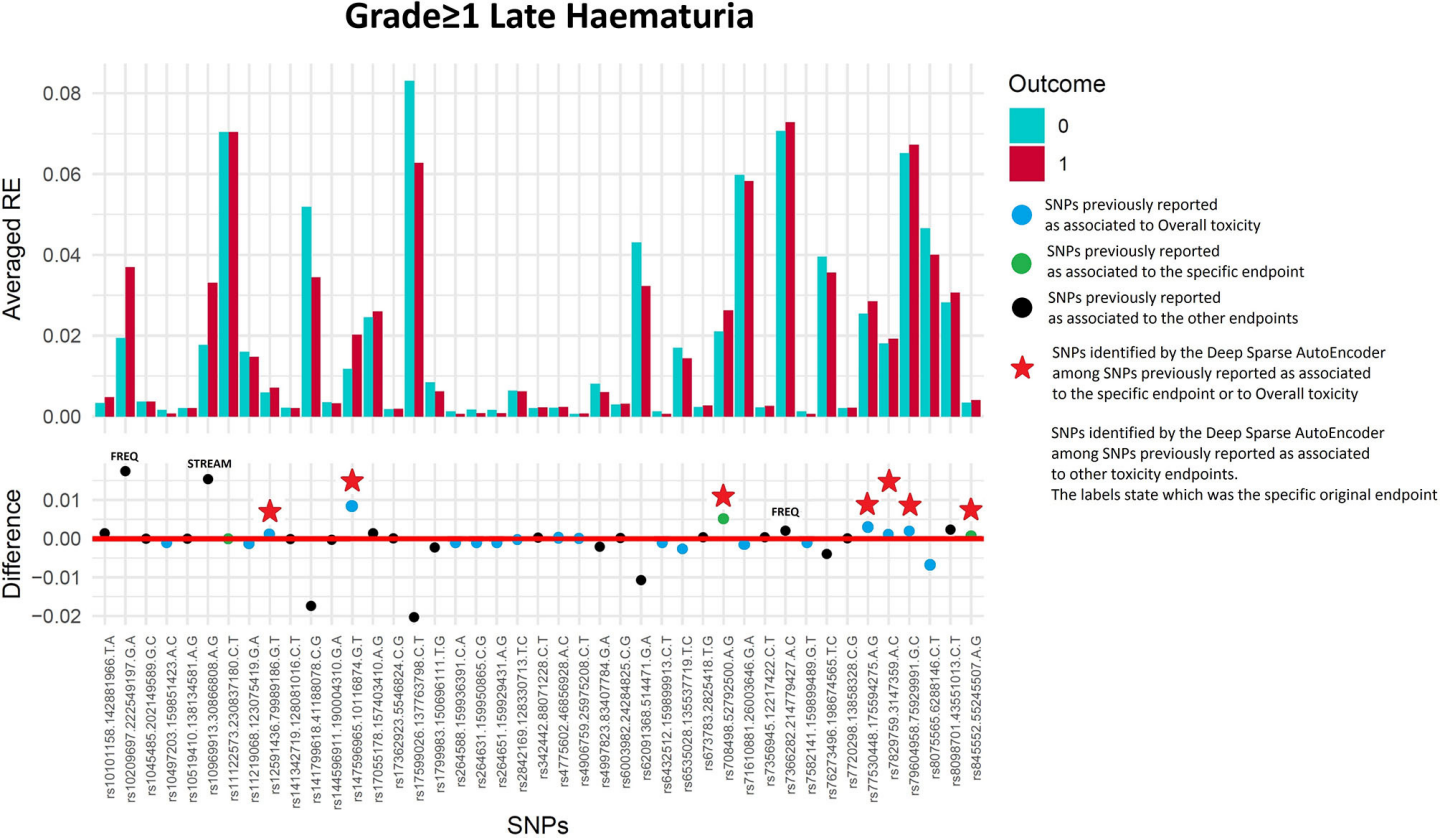
Results for late haematuria grade≥1 from the Deep Sparse AutoEncoder. The 43 considered SNPs are reported in the x-axis and the averaged Reconstruction Errors (RE) are reported in the y-axis (top panel), red columns refer to patients with toxicity, while blue columns refer to patients without toxicity. In the lower panel the difference between averaged Reconstruction Errors between the two classes are represented for each SNP (i.e., differences between red and blue columns). For most SNPs, the difference is close to zero (red line in the bottom panel of the figure). The chosen thresholds in this difference (i.e., highest 30, 20, 10, and 5% differences) are selecting SNPs associated to the toxicity outcome. Green circles refer to SNPs that were previously identified as associated with late haematuria, while blue circles refer to SNPs that were previously associated with overall toxicity as defined by calculation of the Standardized Total Average Toxicity (STAT) score (24). Red stars indicate SNPs (either specific for this endpoint or related to overall toxicity) defining patients with toxicity as outliers with respect to the characteristics of patients without toxicity. Labels show SNPs that not directly associated with late haematuria/overall toxicity, but contributing to their identification. The label states for which toxicity endpoint the SNPs were originally associated with in the literature: BLEE=rectal bleeding, FREQ=urinary frequency, NOCT=nocturia, STREAM=decreased urinary stream.

Seventy-four of 1,343 available patients (5.5%) experienced late haematuria grade≥1. Seventeen patients were excluded from the analysis because they had haematuria at baseline grade≥1, while 41 were excluded because underwent transurethral resection of the bladder or were using anti-muscarinic drugs. Figure 5 and Table 4 report DSAE results for this endpoint: 10 SNPs were identified. Two SNPs already associated with haematuria (*rs708498* and *rs845552*), five SNPs associated with overall toxicity, and three SNPs with other urinary symptoms.

**Table 4:** Results from Deep Sparse AutoEncoder testing of SNPs associated with Late Haematuria^*^.

**SNP**	**References**	**70-th percentile small effect size**	**80-th percentile moderate effect size**	**90-th percentile large effect size**	**95-th percentile large effect size**
**SNPs previously identified as associated to late haematuria**					
rs11122573	(23)	Not validated	Not validated	Not validated	Not validated
rs708498	(22)	**Identified**	**Identified**	**Identified**	Not validated
rs845552	(22)	**Identified**	**Identified**	Not validated	Not validated
**SNPs previously identified as associated to overall toxicity (STAT score)**					
rs147596965	(5)	**Identified**	**Identified**	**Identified**	Not validated
rs77530448	(5)	**Identified**	**Identified**	Not validated	Not validated
rs7829759	(5)	**Identified**	**Identified**	Not validated	Not validated
rs79604958	(5)	**Identified**	**Identified**	Not validated	Not validated
rs12591436	(5)	**Identified**	**Identified**	Not validated	Not validated

* *Late Haematuria grade≥1 (all considered SNPs reported in the table) and to overall toxicity as defined by calculation of the Standardized Total Average Toxicity (STAT) score (24) (in this case only “Identified” SNPs were reported in the table). The SNPs that were correctly identified by the algorithm are flagged as “Identified”*.

#### Late Nocturia Grade≥2

**Figure 6:**
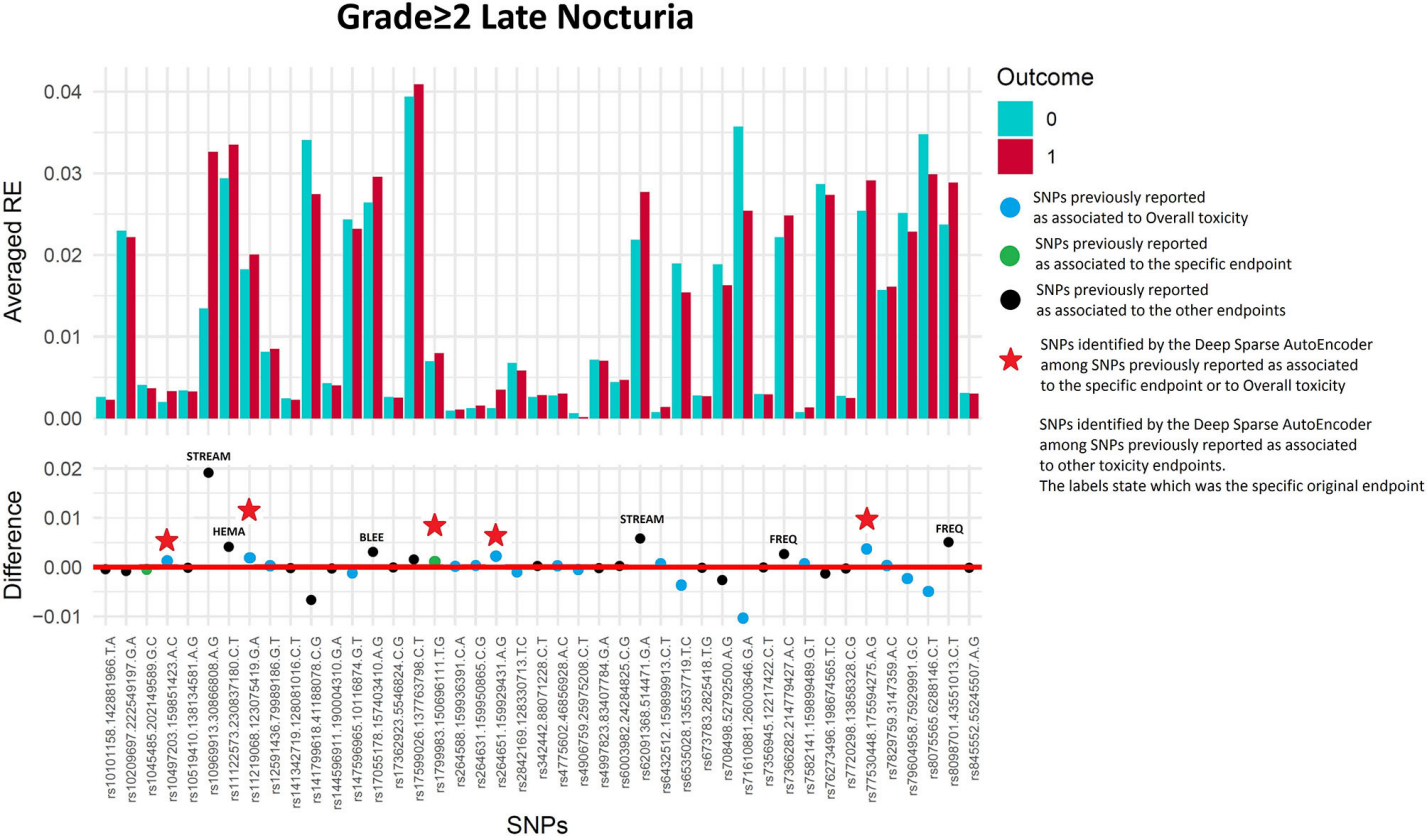
Results for late nocturia grade≥2 from the Deep Sparse AutoEncoder. The 43 considered SNPs are reported in the x-axis and the averaged Reconstruction Errors (RE) are reported in the y-axis (top panel), red columns refer to patients with toxicity, while blue columns refer to patients without toxicity. In the lower panel the difference between averaged Reconstruction Errors between the two classes are represented for each SNP (i.e., differences between red and blue columns). For most SNPs, the difference is close to zero (red line in the bottom panel of the figure). The chosen thresholds in this difference (i.e., highest 30, 20, 10, and 5% differences) are selecting SNPs associated to the toxicity outcome. Green circles refer to SNPs that were previously identified as associated with late nocturia, while blue circles refer to SNPs that were previously associated with overall toxicity as defined by calculation of the Standardized Total Average Toxicity (STAT) score (24). Red stars indicate SNPs (either specific for this endpoint or related to overall toxicity) defining patients with toxicity as outliers with respect to the characteristics of patients without toxicity. Labels show SNPs that not directly associated with late nocturia/overall toxicity, but contributing to their identification. The label states for which toxicity endpoint the SNPs were originally associated with in the literature: BLEE=rectal bleeding, FREQ=urinary frequency, HEMA=haematuria, STREAM=decreased urinary stream.

Two hundred and twenty-three patients out of 1,250 available patients (17.8%) experienced late nocturia grade≥2. One hundred and ten patients were excluded from analysis because they had nocturia grade≥2 at baseline, while 41 were excluded because underwent transurethral resection of the bladder or were using anti-muscarinic drugs. Figure 6 and Table 5 report results for the validation through DSAE in the REQUITE population. Eleven SNPs were identified: one SNP already found to be associated with nocturia, four with overall toxicity, one with bleeding and five with other urinary symptoms.

**Table 5:** Results from Deep Sparse AutoEncoder testing of SNPs associated with Late Nocturia^*^.

**SNP**	**References**	**70-th percentile small effect size**	**80-th percentile moderate effect size**	**90-th percentile large effect size**	**95-th percentile large effect size**
**SNPs previously identified as associated to late nocturia**					
rs1799983	(22)	**Identified**	Not validated	Not validated	Not validated
rs1045485	(22)	Not validated	Not validated	Not validated	Not validated
**SNPs previously identified as associated to overall toxicity (STAT score)**					
rs10497203	(11)	**Identified**	**Identified**	Not validated	Not validated
rs264651	(11)	**Identified**	**Identified**	Not validated	Not validated
rs77530448	(5)	**Identified**	**Identified**	Not validated	Not validated
rs11219068	(5)	**Identified**	**Identified**	Not validated	Not validated

* *Late Nocturia grade≥2 (all considered SNPs reported in the table) and to overall toxicity as defined by calculation of the Standardized Total Average Toxicity (STAT) score (24) (in this case only “Identified” SNPs were reported in the table). The SNPs that were correctly identified by the algorithm are flagged as “Identified”*.

#### Late Decreased Urinary Stream Grade≥1

**Figure 7:**
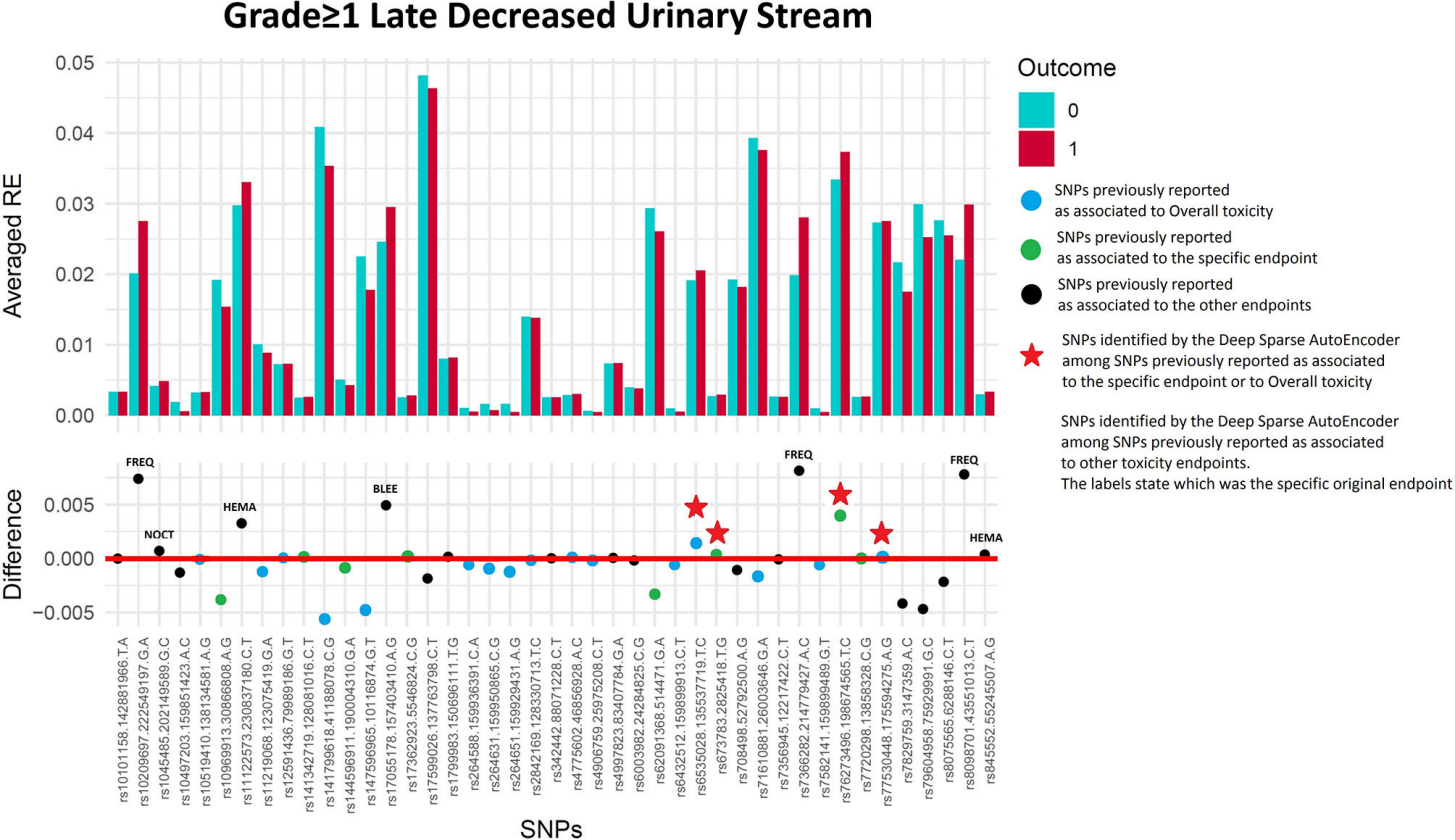
Results for late decreased urinary stream grade≥1 from the Deep Sparse AutoEncoder. The 43 considered SNPs are reported in the x-axis and the averaged Reconstruction Errors (RE) are reported in the y-axis (top panel), red columns refer to patients with toxicity, while blue columns refer to patients without toxicity. In the lower panel the difference between averaged Reconstruction Errors between the two classes are represented for each SNP (i.e., differences between red and blue columns). For most SNPs, the difference is close to zero (red line in the bottom panel of the figure). The chosen thresholds in this difference (i.e., highest 30, 20, 10, and 5% differences) are selecting SNPs associated to the toxicity outcome. Green circles refer to SNPs that were previously identified as associated with late decreased urinary stream, while blue circles refer to SNPs that were previously associated with overall toxicity as defined by calculation of the Standardized Total Average Toxicity (STAT) score (24). Red stars indicate SNPs (either specific for this endpoint or related to overall toxicity) defining patients with toxicity as outliers with respect to the characteristics of patients without toxicity. Labels show SNPs that not directly associated with late decreased urinary stream /overall toxicity, but contributing to their identification. The label states for which toxicity endpoint the SNPs were originally associated with in the literature: BLEE=rectal bleeding FREQ=urinary frequency, HEMA=haematuria, NOCT=nocturia.

Two hundred and eleven out of 1,234 available patients (17.1%) experienced late decreased stream grade≥1. One hundred and twenty-six patients were excluded from analysis because they had decreased stream grade≥1 at baseline, while 41 were excluded because underwent transurethral resection of the bladder or were using anti-muscarinic drugs. Eleven SNPs were selected: two SNPs previously identified for decreased urinary stream (*rs76273496* and *rs673783*), two for overall toxicity, six for other urinary symptoms and one for bleeding (Figure 7 and Table 6).

**Table 6:** Results from Deep Sparse AutoEncoder testing of SNPs associated with Late Decreased Urinary Stream^*^.

**SNP**	**References**	**70-th percentile small effect size**	**80-th percentile moderate effect size**	**90-th percentile large effect size**	**95-th percentile large effect size**
**SNPs previously identified as associated to late decreased urinary stream**					
rs7720298	(5)	Not validated	Not validated	Not validated	Not validated
rs17362923	(5)	Not validated	Not validated	Not validated	Not validated
rs76273496	(5)	**Identified**	**Identified**	**Identified**	Not validated
rs144596911	(5)	Not validated	Not validated	Not validated	Not validated
rs62091368	(5)	Not validated	Not validated	Not validated	Not validated
rs141342719	(5)	Not validated	Not validated	Not validated	Not validated
rs673783	(5)	**Identified**	Not validated	Not validated	Not validated
rs10969913	(23)	Not validated	Not validated	Not validated	Not validated
**SNPs previously identified as associated to overall toxicity (STAT score)**					
rs77530448	(5)	**Identified**	Not validated	Not validated	Not validated
rs6535028	(5)	**Identified**	Not validated	Not validated	Not validated

* *Late Decreased Urinary Stream grade≥1 (all considered SNPs reported in the table) and to overall toxicity as defined by calculation of the Standardized Total Average Toxicity (STAT) score (24) (in this case only “Identified” SNPs were reported in the table). The SNPs that were correctly identified by the algorithm are flagged as “Identified”*.

### Classical Validation Approach Using Univariate Analysis

A simple validation approach, using univariate logistic analysis, identified eight SNPs with *p* < 0.05 (range 0.01–0.05), none of them is validated when considering the Bonferroni correction for multiple testing, which would require *p* < 0.0011 in this case. Detailed results are presented in Supplementary Table 4.

## Discussion

In recent years Normal Tissue Complication Probability (NTCP) models have been developed to attempt to predict before the start of treatment patients at risk of long-term radiation toxicity. These recent developments were also characterized by the shift from NTCP dose-based modeling to the wider field of more “comprehensive” predictive models. In the speculative case that two patients receive exactly the “same dose distribution,” the risk of toxicity is always modulated by the single individual profile.

The fact that “dose is not enough” was clear from the early days of radiobiology but is receiving constantly growing attention in the current “omics” epoch (Bentzen, 2006): the availability of individual information characterizing patients and potentially influencing their reactions to radiation is increasingly important, especially in the era of image-guided radiotherapy that can spare the organs at risk in most patients.

The purpose of any predictive model in oncology is to provide valid outcome predictions for new patients. Essentially, the main interest of a dataset used to develop a model is to learn for the future. Systematic validation in multi-center collaborative settings hence is a crucial aspect in the process of predictive modeling. REQUITE is the largest multi-center observational study in this field to date, collecting standardized data longitudinally. The study was specifically designed to enable validation of models and biomarkers that predict a patient's risk of developing long-term side-effects following radiotherapy.

The present work focused on the validation of findings from previous GWAS of radiation toxicity after radiotherapy for prostate cancer. To the best of our knowledge, few validation studies in this frame have been conducted so far. Barnett et al. (13) performed an independent validation study of 92 SNPs in 46 genes in a large cohort of breast (976 patients) and prostate (637 patients) cancer patients who received radiotherapy. They focused on five rectal (bleeding, proctitis, sphincter control, stool frequency, tenesmus) and four urinary endpoints (frequency, nocturia, incontinence, and decreased stream) reported by patients 2 years after radiotherapy. An additional endpoint of overall toxicity as measured by the STAT score was also considered. None of the investigated associations was confirmed after adjustment for multiple comparisons.

Genome-wide radiogenomic studies are identifying and validating SNPs. However, to date these studies have relied on the classical single marker association test (both in the discovery and validation setting), which is hampered by the need for multiple-testing corrections. For typical study sizes, this method can detect only relatively large effect size and has limited power to identify reliably modest effects from the many SNPs that are likely to contribute to a polygenic risk profile associated with radiation toxicity. Genome-wide studies miss SNPs that make small but real contributions to risk.

Machine learning has already been proposed as a promising alternative approach to estimate overall genetic risk (27). The approach can identify multiple SNPs with small effects that together but not individually reach genome-wide significance. Two studies have already proposed machine learning methods to identify SNP-based signatures associated with late toxicity after radiotherapy for prostate cancer (27, 28).

Here, we extended the use of machine learning methods by using a method that addresses an important limitation of studies on radiation toxicity: the imbalance of classes, with a lower frequency of patients *with* vs. *without* late toxicity. This imbalance is important because it can lead to sub-optimal solutions (29), even when datasets are used for validation. As a first step in testing our approach, we attempted to and were successful in validating previously reported associations identified in studies based on classical single marker association tests. The next step will be a *de novo* analysis to identify SNPs with smaller individual effects.

Dealing with imbalance requires non-classical statistical solutions. Here, we explore novel methods for feature selection that come from the Deep Learning research field (25). Indeed, deep learning approaches, with their intrinsic hierarchical structure (where each layer realizing a combination of the previous layer), seem particularly adept at mimicking complex dependencies within data. Deep learning has already been applied and shown to have potential in similar bioinformatics research areas, such as for modeling the competition between splice sites (30) and in predicting RNA- and DNA-binding specificity (31).

We used DSAE to obtain the best possible representation of the majority class (without toxicity) and so to identify which features (SNPs) distinguish the minority class (with toxicity). The encoder and decoder functions are usually non-linear (i.e., sigmoid, hyperbolic tangent, rectified linear unit etc.), which enables a better reconstruction of the input by the capture of complex non-linear relationships among SNPs. Training on healthy patients allows the overall SNP pattern of normal radio-sensitivity to be established. Testing measures the “distance” between each new patient and the pattern of normal radio-sensitivity to identify SNPs associated with the highest reconstruction errors (i.e., highest distances) between the pattern of normality and the SNP profile of patients scored with toxicity (i.e., radio-sensitive patients). The distribution of the reconstructed errors allows identification and classification of SNPs with very large/large effect (SNPs associated with the top 95th percentile and 90th percentile of the distribution of reconstructed errors) and with moderate/small effects (SNPs associated with the top 80th percentile and 70th percentile of the distribution of reconstructed errors).

The DSAE successfully validated multiple SNPs contributing to an increased risk of toxicity. Some SNPs were already associated with the specific considered endpoint, others were previously associated with overall toxicity, and some were previously associated with other toxicities.

As common in GWAS, many significant SNPs lie in non-coding regions, and it is premature to speculate on their functional significance. We refer readers to the original publications which discuss possible gene functions (5, 11, 23), but give an example to illustrate likely clinical relevance. DSAE validated two SNPs previously associated with haematuria, *rs708498* and *rs845552*, which are located in the *PTGER2* and *EGFR* genes, respectively. *PTGER2* (widely distributed in humans) encodes Prostaglandin E2 receptor 2. Irradiation causes hypermethylation of this antifibrotic gene (32). *EGFR* has been shown to play a critical role in TGF-β1 dependent fibroblast to myofibroblast differentiation (33). These two SNPs were also identified for urinary stream (*rs845552*) and urinary frequency (*rs708498*).

The main strength of our study is use of a large international prospective multi-center cohort of patients treated with modern radiotherapy techniques and fractionation schemes. The patients were specifically enrolled to validate models and biomarkers for predicting radiation toxicity, and the study design involved a standardized data collection scheme for collecting healthcare professional and patient-reported outcomes. The extensive role of data management also allowed for quality assurance of data collected, and we used “real world” data coming from “data-farming” (34).

A possible limitation of our study was use of 2-year follow-up toxicity data. The REQUITE study is still maturing, normal tissue reactions in the intestinal and urinary tract develop gradually from 6 months after radiotherapy till to around 3 years for the intestinal syndrome and to 5 years for the urinary syndrome. Recent additional funding is allowing extension of the REQUITE study with the aim of reaching standardized collection of follow-up data till year 5.

The use of grade 1 and grade 2 events is another possible limitation of this study. As the application of deep learning techniques requires a suitable number of events, the choice of mild or moderate (when possible) toxicity was forced by the number of morbidity events registered in the REQUITE population. The low number of severe toxicity is for sure a reflection of modern radiotherapy techniques which allow a substantial sparing of normal tissues, at least for the case of prostate cancer irradiation. Yet, some grade 1 and grade 2 toxicity can assume a chronic behavior, with substantial impact on the quality of life of long term survivors, for example, this could happen, for grade 2 urinary frequency and nocturia which are impairing daily activities and the quality of sleep for many years (35). A further point, more associated to research rather to clinical activity, is related to the possibility that the same genes/variants predispose to severe toxicity that predispose to low-grade toxicity. A realistic hypothesis is that some genes/variants will be common and others will be unique to severe toxicities. For example, ATM seems to be important for both mild and severe toxicity, though the particular variants differ with common SNPs associated with any toxicity, but rare mutations associated with severe toxicity. We think we can make a good case that genes identified via GWAS of mild toxicity represent good candidates for subsequent sequencing studies to identify rare mutations that may be associated with severe toxicities. Probably there are at least some biologic mechanisms common to both mild and severe toxicity, though the optimal genomic signature for each may differ. Our work still adds value by pointing to the candidate genes or loci that are likely important for both.

We have shown our approach is worth studying further and the next step would be to use it to identify patterns of SNPs to define polygenic risk scores that can be included into integrated normal tissue complication probability models, together with validated dosimetric and clinical risk factors.

The DSAE methodology underlines that, within the current RT, experiencing no toxicity could be considered as the “normal” situation, with patients with mild/moderate toxicity being outliers. The possible knowledge of the single patient intrinsic radiosensitivity and the identification of these outlier subjects could help in tailoring decision making. This should not entail changing the probability of tumor control to avoid mild/moderate side-effects, yet it should be focused on maximizing uncomplicated tumor control, even considering the patient inclination toward the different side-effects. The availability of such models would be relevant for the clinic, allowing the single patient optimization, thus constituting an important step toward the implementation of predictive modeling in the clinic. This approach would allow tailoring of therapeutic approach (i.e., active surveillance vs. prostatectomy vs. brachytherapy vs. external beam radiotherapy) and of doses (both to tumor and organs at risk) to the specific patient anatomy, clinical situation and individual biology. Combining biological stratification with toxicity reducing techniques (such as imaging fusion, image guidance, fractionation and reduced margins for Planning Target Volume) could further decrease treatment related toxicity rates and allow for dose escalation to enhance tumor control. Integrated predictive models will also be an essential tool in the design of interventional trials to modify the radiotherapy strategies. A detailed discussion of the potential ways in which biomarker/SNP assays might be implemented in routine clinical practice can be found in Azria et al. (7).

Other future work could study the possibility of “scaling” the use of DSEAs to the discovery of new genetic signatures using the whole GWAS information available in the REQUITE population, thus achieving the possibility of considering millions of features to detect outliers.

## Conclusion

A deep learning approach can validate SNPs associated with toxicity after radiotherapy. The method can identify complex SNP signatures for multiple toxicity endpoints and should be studied further to extract polygenic risk scores to include in integrated normal tissue complication probability models that could be used to personalize radiotherapy planning.

## Data Availability Statement

Funding for the five year REQUITE project ended on 30th September 2018. REQUITE does not benefit financially from supplying data and/or samples to researchers, but does make a charge to cover its costs and support continued maintenance of the database and biobank beyond the ending of the funding period. To facilitate this continued access to researchers, the REQUITE Steering Committee approved a tiered cost recovery model for access to data and/ or samples. Contact REQUITE (requite@manchester.ac.uk) for more information on pricing.

## Ethics Statement

The REQUITE study was reviewed and approved by North West - Great Manchester East Ethics Committee (UK, reference 14 NW 0035) and by the local Ethics Committees of all participating centers. The patients provided their written informed consent to participate in this study and for the publication of the data included in this article.

## Author Contributions

MM, AP, FG, TRan, and CW: study design. MM, FG, and TRan: study development. AP, FI, AM, PZ, RE, and JC-C: coordination/supervision of the study. LV, PO, VF, TRat, PRS, KJ, ML, KH, GdM, DdR, BV, EvL, ACh, ES, CH, MV, BA, RV, DA, M-PJ, RS, KC, and PC: patient enrolment and follow-up. CT, TG, ACi, BR, AV, and MA-B: collection of the data. LF, AD, SK, and DP: SNP assay. JC-C, PS, AW, and RE: trial and data management. MM, FG, NF, FI, AP, AM, and TRan: statistical analysis. MM, FG, NF, TRan, and CW: draft of the paper. All authors: critical revision of the manuscript/final approval.

## Conflict of Interest

The authors declare that the research was conducted in the absence of any commercial or financial relationships that could be construed as a potential conflict of interest.
