# Supplementary material for: A Deep Learning Approach Validates Genetic Risk Factors for Late Toxicity After Prostate Cancer Radiotherapy in a REQUITE Multi-National Cohort
Source: Front Oncol. 2020 Oct 15;10:541281. doi: 10.3389/fonc.2020.541281 (PMC7593843; doi:10.3389/fonc.2020.541281)
Supplement: Supplementary file 1 [file Data_Sheet_1.docx]

Supplementary Material

**Figure S1.** Correlation matrix for the selected 43 SNPs in the Requite population. Of note the only correlated SNPs are six variants already known to be highly correlated in European population and to represent the same association signal (Fachal et al., 2014) (correlation coefficients between 0.96 and 0.99 in these cases).

**
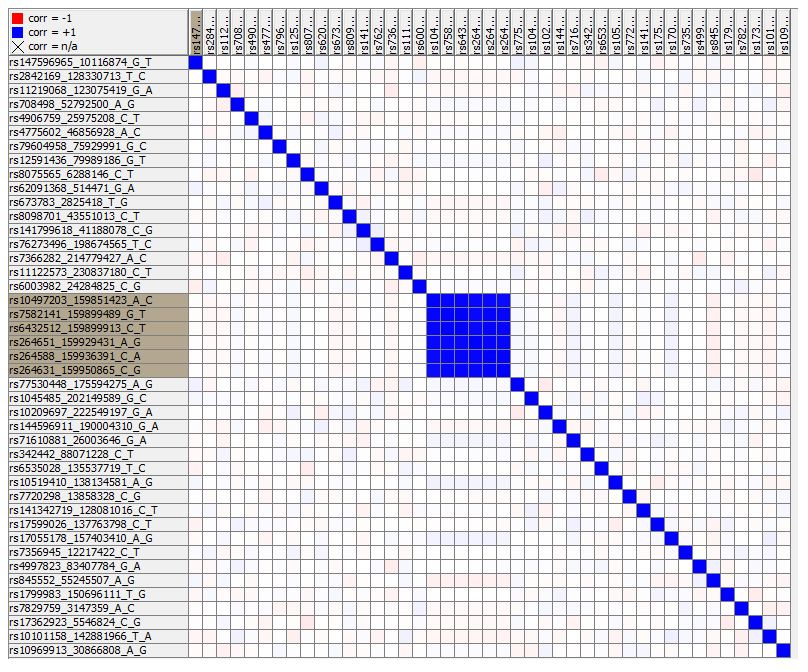
**

**Table S1**. Clinical characteristics of the cohort for the late rectal bleeding outcome. For continuous variables (i.e., age, BMI and Equivalent Uniform Dose, EUD) mean value and standard deviation are reported. The other variables are categorical and the number of patients that showed that specific condition is reported together with percentage in brackets.

N=number; BMI=body mass index; TURP=Transurethral resection of the prostate; EUD=Equivalent Uniform Dose calculated with volume parameter n=0.12; RT=radiotherapy; 3DCRT=three-dimensional conformal radiotherapy; IMRT=Intensity Modulated Radiation Therapy; VMAT= Volumetric Modulated Radiation Therapy

|  | **Grade≥1**  **rectal bleeding^1^** |
| --- | --- |
| **Total N** | **1366** |
| **Patients with outcome** | **160 (11.7%)** |
| Age (years) | 69.4 (6.9) |
| BMI^∗^ (kg/m^2^) | 27.6 (4.2) |
| Smoke^∗^ (never) | 513 (37.8%) |
| Smoke^∗^ (stop before diagnosis) | 647 (47.6%) |
| Smoke^∗^ (stop at diagnosis) | 38 (2.8%) |
| Smoke^∗^ (current) | 161 (11.9%) |
| Alcohol^∗^ (never) | 166 (12.3%) |
| Alcohol^∗^ (stop before diagnosis) | 98 (7.3%) |
| Alcohol^∗^ (stop after diagnosis) | 36 (2.7%) |
| Alcohol^∗^ (current) | 1051 (77.8%) |
| Diabetes | 172 (12.6%) |
| Hypertension | 795 (58.2%) |
| Other cardiovascular diseases | 324 (23.7%) |
| Use of lipid lowering drugs | 513 (37.6%) |
| No inflammatory bowel disease | 1296 (94.9%) |
| Crohn’s disease | 14 (1.0%) |
| Colitis ulcerosa | 11 (0.8%) |
| Diverticulitis | 37 (2.7%) |
| Other inflammatory bowel diseases | 8 (0.6%) |
| Use of 5alpha reductase inhibitors or of alpha blockers | 164 (12.0%) |
| Transurethral resection of the Bladder | 29 (2.1%) |
| No abdominal surgery | 893 (65.6%) |
| Appendectomy | 145 (10.7%) |
| Cholecystectomy | 44 (3.2%) |
| Rectum-sigma resection | 12 (0.9%) |
| Nephrectomy | 6 (0.4%) |
| Other | 261 (19.2%) |
| TURP | 103 (7.5%) |
| Radical prostatectomy | 415 (30.4%) |
| Hormone therapy | 979 (71.7%) |
| Irradiation of pelvic lymph nodes | 437 (32.0%) |
| Irradiation of seminal vesicles^∗^ | 793 (58.1%) |
| 3DCRT | 248 (18.2%) |
| Static field IMRT | 157 (11.5%) |
| VMAT/Rapid Arc | 961 (70.4%) |
| Rectal EUD^∗^ (n=0.12, Gy) | 58.8 (7.5) |
| ^1^ Grade≥1 late rectal bleeding as defined in the main text: patients exhibiting at least mild bleeding (even requiring no intervention) at 12 or at 24 months. Patients with grade≥1 at baseline and grade≤1 during follow-up were considered as not bleeders; patients with haemorrhoids before radiotherapy treatment were excluded.  ^∗^ Missing values: 5 for BMI, 3 for EUD, 7 for Smoking habits, 15 for alcohol habits and 22 for irradiation of seminal vesicles. | |

**Table S2**. Clinical characteristics of the cohort for the late urinary symptom outcomes. For continuous variables (i.e., age, BMI) mean value and standard deviation are reported. The other variables are categorical and the number of patients that showed that specific condition is reported together with percentage in brackets.

N=number; BMI=body mass index; TURP=Transurethral resection of the prostate; RT=radiotherapy; 3DCRT=three-dimensional conformal radiotherapy; IMRT=Intensity Modulated Radiation Therapy; VMAT= Volumetric Modulated Radiation Therapy

|  | **Grade≥2**  **urinary frequency^1^** | **Grade≥1**  **Haematuria^2^** | **Grade≥2**  **Nocturia^3^** | **Grade≥1**  **Decreased stream^4^** |
| --- | --- | --- | --- | --- |
| **Total N** | **1334** | **1343** | **1250** | **1234** |
| **Patients with outcome** | **56 (4.2%)** | **74 (5.5%)** | **223 (17.8%)** | **211 (17.1%)** |
| Age (years) | 69.3 (6.9) | 69.3 (6.9) | 69.3 (6.9) | 69.3(6.8) |
| BMI^∗^ (kg/m^2^) | 27.6 (4.2) | 27.6 (4.2) | 27.6 (4.2) | 27.6 (4.2) |
| Smoke^∗^ (never) | 501 (37.8 %) | 505 (37.8%) | 468 (37.6%) | 461 (37.5%) |
| Smoke^∗^ (stop before diagnosis) | 629 (47.4%) | 633 (47.4%) | 592 (47.6%) | 586 (47.7%) |
| Smoke^∗^ (stop after diagnosis) | 36 (2.7%) | 37 (2.8%) | 35 (2.8%) | 35 (2.9%) |
| Smoke^∗^ (current) | 161 (12.1%) | 161 (12.1%) | 149 (12.0%) | 146 (11.9%) |
| Alcohol^∗^ (never) | 161 (12.2%) | 162 (12.2%) | 144 (11.6%) | 143 (11.7%) |
| Alcohol^∗^ (stop before diagnosis) | 97 (7.4%) | 97 (7.3%) | 89 (7.2%) | 87 (7.1%) |
| Alcohol^∗^ (stop after diagnosis) | 34 (2.6%) | 34 (2.6%) | 30 (2.4%) | 30 (2.5%) |
| Alcohol^∗^ (current) | 1028 (77.9%) | 1035 (77.9%) | 975 (78.8%) | 961 (78.7%) |
| Diabetes | 167 (12.5%) | 169 (12.6%) | 156 (12.5%) | 156 (12.6%) |
| Hypertension | 773 (58.0%) | 780 (58.1%) | 716 (57.3%) | 705 (57.1%) |
| Other cardiovascular disease | 321 (24.1%) | 322 (24.0%) | 301 (24.1%) | 296 (24.0%) |
| Use of lipid lowering drugs | 502 (37.6%) | 504 (37.5%) | 472 (37.8%) | 468 (37.9%) |
| No inflammatory bowel disease | 1264 (94.8%) | 1272 (94.7%) | 1180 (94.4 %) | 1166 (94.5%) |
| Crohn’s disease | 13 (1.0%) | 14 (1.0%) | 13 (1.0%) | 13 (1.1%) |
| Colitis ulcerosa | 11 (0.8%) | 11 (0.8%) | 10 (0.8%) | 10 (0.8%) |
| Diverticulitis | 38 (2.9%) | 38 (2.8%) | 38 (3.0%) | 37 (3.0%) |
| Other inflammatory bowel diseases | 8 (0.6%) | 8 (0.6%) | 9 (0.7%) | 8 (0.7%) |
| Use of 5 alpha reductase inhibitors or of alpha blockers | 164 (12.3%) | 165 (12.3%) | 152 (12.2%) | 153 (12.4%) |
| No abdominal surgery | 872 (65.6%) | 879 (65.7%) | 812 (65.2%) | 803 (65.3%) |
| Appendectomy | 143 (10.8%) | 143 (10.7%) | 135 (10.8%) | 132 (10.7%) |
| Cholecystectomy | 41 (3.1%) | 41 (3.1%) | 37 (3.0%) | 35 (2.9%) |
| Rectum-sigma resection | 11 (0.8%) | 11 (0.8%) | 9 (0.7%) | 9 (0.7%) |
| Nephrectomy | 6 (0.5%) | 6 (0.5%) | 6 (0.5%) | 6 (0.5%) |
| Other | 256 (19.2%) | 258(19.2%) | 247(19.7%) | 245 (19.8%) |
| TURP | 87 (6.5%) | 88 (6.6%) | 81 (6.5%) | 79 (6.4%) |
| Radical prostatectomy | 409 (30.7%) | 412 (30.7%) | 390 (31.2%) | 386 (31.3%) |
| Hormone therapy | 955 (71.6%) | 963 (71.7%) | 892 (71.4%) | 878 (71.2%) |
| Irradiation of pelvic lymph nodes | 423 (31.7%) | 429 (31.9%) | 395 (31.6%) | 388 (31.4%) |
| Irradiation of seminal vesicles^∗^ | 772 (57.9%) | 778 (57.9%) | 726 (58.1%) | 715 (57.9%) |
| 3DCRT | 242 (18.1%) | 242 (18.0%) | 229 (18.3%) | 226 (18.3%) |
| Static field IMRT | 156 (11.7%) | 156 (11.6%) | 143 (11.4%) | 139 (11.3%) |
| VMAT/Rapid arc | 936 (70.2%) | 945 (70.4%) | 878 (70.2%) | 869 (70.4%) |
| Maximum dose to the Bladder (α/β=1.5 Gy, Gy) | 73.6 (4.3) | 73.6 (4.3) | 73.6 (4.3) | 73.6 (4.3) |
| Maximum dose to the Bladder (α/β=3 Gy, Gy) | 73.0 (4.5) | 73.0 (4.5) | 73.0 (4.5) | 73.0 (4.5) |
| ^1^Grade≥2 late urinary frequency defined as described in the main text: patients with urinary frequency limiting instrumental activities of daily living or if medical management at 12 OR at 24 months. Patients with grade≥2 urinary frequency at baseline were considered as not exhibiting this endpoint.  ^2^Grade ≥1 late haematuria defined as described in the main text: patients with asymptomatic haematuria (clinical or diagnostic observations only, no intervention indicated) at 12 OR 24 months. Patients with grade≥1 haematuria at baseline and grade≤1 during follow-up were considered as not exhibiting the endpoint.  ^3^Grade ≥2 late nocturia defined as described in the main text: patients declaring need to urinate at least two-three times per night at 12 OR 24 months. Patients with grade≥2 nocturia at baseline and grade≤2 during follow-up were considered as not exhibiting the endpoint.  ^4^Grade≥1 late decreased urinary stream defined as described in the main text: patients scored with hesitant or dripping stream at 12 OR 24 months. Patients with grade≥1 haematuria at baseline and grade≤1 during follow-up were considered as not exhibiting the endpoint.  ^∗^ Missing values; for the urinary frequency endpoint: 5 BMI, 7 Smoking habits, 14 Alcohol habits and 23 irradiation of seminal vesicles; for the haematuria endpoint:, 5 BMI, 7 Smoking habits, 15 Alcohol habits and 23 irradiation of seminal vesicles; for the nocturia endpoint:4 BMI, 6 Smoking habits, 12 Alcohol habits and 20 irradiation of seminal vesicles ; for decreased urinary flow: 4 BMI, 6 Smoking habits, 13 Alcohol habits and 20 irradiation of seminal vesicles. | | | | |

**Table S3**. Summary of SNPs identified by the Deep Sparse AutoEncoder as classifying patients with toxicity as outliers with respect to the characteristics (in terms of SNP profile) of patients without toxicity. The toxicity endpoint reported in bold identifies the endpoint for which the association was found in the original reference. In italics results for SNPs associated to overall toxicity.

bleeding=late rectal bleeding; frequency=urinary frequency; stream=decreased urinary stream

| **SNP** | **Reference** | **bleeding** | **nocturia** | **frequency** | **stream** | **haematuria** | |
| --- | --- | --- | --- | --- | --- | --- | --- |
| *rs17055178* | Kerns et al. 2020 |  | nocturia | frequency | stream |  | |
| *rs17599026* | Kerns et al. 2016 | bleeding |  | **frequency** |  |  | |
| *rs8098701* | Kerns et al. 2016 | bleeding | nocturia | **frequency** | stream |  | |
| *rs7366282* | Kerns et al. 2016 |  | nocturia | **frequency** | stream | haematuria | |
| *rs10209697* | Kerns et al. 2016 |  |  | **frequency** | stream | haematuria | |
| *rs76273496* | Kerns et al. 2016 | bleeding |  | frequency | **stream** |  | |
| *rs673783* | Kerns et al. 2016 |  |  |  | **stream** |  | |
| *rs10969913* | Kerns et al. 2020 | bleeding | nocturia | frequency |  | haematuria | |
| *rs11122573* | Kerns et al. 2020 | bleeding | nocturia | frequency | stream |  | |
| *rs708498* | De Langhe et al. 2014 |  |  | frequency |  | **haematuria** | |
| *rs845552* | De Langhe et al. 2014 |  |  |  | stream | **haematuria** | |
| *rs1799983* | De Langhe et al. 2014 |  | **nocturia** | frequency |  |  | |
| *rs1045485* | De Langhe et al. 2014 |  |  |  | stream |  | |
| *rs10497203* | Fachal et al. 2014 |  | *nocturia* |  |  |  |  |
| *rs264651* | Fachal et al. 2014 |  | *nocturia* |  |  |  |  |
| *rs264631* | Fachal et al. 2014 | *bleeding* | *nocturia* |  |  |  |  |
| *rs147596965* | Kerns et al. 2016 |  |  | *frequency* |  | *haematuria* |  |
| *rs77530448* | Kerns et al. 2016 |  | *nocturia* | *frequency* | *stream* | *haematuria* |  |
| *rs141799618* | Kerns et al. 2016 | *bleeding* |  |  |  |  |  |
| *rs11219068* | Kerns et al. 2016 |  | *nocturia* |  |  |  |  |
| *rs8075565* | Kerns et al. 2016 |  |  | *frequency* |  |  |  |
| *rs6535028* | Kerns et al. 2016 |  |  |  | *stream* |  |  |
| *rs7829759* | Kerns et al. 2016 |  |  |  |  | *haematuria* |  |
| *rs79604958* | Kerns et al. 2016 |  |  |  |  | *haematuria* |  |
| *rs12591436* | Kerns et al. 2016 |  |  | *frequency* |  | *haematuria* |  |

**Table S4**. Summary of results association between SNPs and the selected toxicity endpoints when using logistic regression. P-value≤0.05 are highlighted in bold. Of note the Bonferroni correction for multiple testing would require a p-value≤0.001 to be statistically significant. bleeding=late rectal bleeding; frequency=urinary frequency; stream=decreased urinary stream.

|  | **previous association** | **REFERENCE** | **bleeding** | **haematuria** | **frequency** | **stream** | **nocturia** |
| --- | --- | --- | --- | --- | --- | --- | --- |
| *rs10519410* | bleeding | Kerns et al. 2013 | 0.56 | 0.33 | 0.17 | 0.80 | 0.89 |
| *rs17055178* | bleeding | Kerns et al. 2020 | 0.86 | 0.14 | 0.46 | 0.92 | 0.29 |
| *rs10101158* | frequency | Kerns et al. 2016 | 0.89 | 0.28 | 0.70 | 0.78 | **0.02** |
| *rs10209697* | frequency | Kerns et al. 2016 | 0.82 | 0.56 | 0.86 | **0.05** | 0.41 |
| *rs17599026* | frequency | Kerns et al. 2016 | 0.62 | 0.42 | 0.61 | 0.68 | 0.34 |
| *rs342442* | frequency | Kerns et al. 2016 | 0.63 | 0.58 | 0.79 | 0.16 | 0.14 |
| *rs4997823* | frequency | Kerns et al. 2016 | 0.78 | 0.15 | 0.44 | 0.47 | 0.20 |
| *rs6003982* | frequency | Kerns et al. 2016 | 0.61 | 0.37 | 0.63 | 0.69 | 0.44 |
| *rs7356945* | frequency | Kerns et al. 2016 | **0.01** | 0.75 | 0.47 | 0.96 | 0.61 |
| *rs7366282* | frequency | Kerns et al. 2016 | 0.99 | 0.84 | **0.05** | 0.41 | 0.85 |
| *rs8098701* | frequency | Kerns et al. 2016 | 0.87 | 0.87 | 0.48 | 0.06 | 0.55 |
| *rs11122573* | haematuria | Kerns et al. 2020 | 0.18 | 0.78 | 0.50 | 0.78 | 0.48 |
| *rs708498* | haematuria | De Langhe et al. 2014 | 0.11 | 0.30 | 0.93 | 0.10 | 0.69 |
| *rs845552* | haematuria | De Langhe et al. 2014 | 0.76 | 0.14 | 0.44 | 0.97 | 0.95 |
| *rs1045485* | nocturia | De Langhe et al. 2014 | 0.97 | 0.68 | 0.48 | 0.96 | 0.82 |
| *rs1799983* | nocturia | De Langhe et al. 2014 | 0.68 | 0.92 | 0.33 | 0.30 | 0.22 |
| *rs10969913* | stream | Kerns et al. 2020 | 0.69 | 0.49 | 0.40 | 0.64 | **0.01** |
| *rs141342719* | stream | Kerns et al. 2016 | 0.75 | 0.10 | 0.12 | 0.72 | 0.49 |
| *rs144596911* | stream | Kerns et al. 2016 | 0.22 | 0.06 | 0.52 | 0.07 | 0.60 |
| *rs17362923* | stream | Kerns et al. 2016 | 0.84 | 0.48 | 0.48 | 0.27 | 0.87 |
| *rs62091368* | stream | Kerns et al. 2016 | 0.88 | 0.13 | 0.36 | **0.04** | 0.46 |
| *rs673783* | stream | Kerns et al. 2016 | 0.61 | 0.46 | 0.13 | 0.97 | 0.20 |
| *rs76273496* | stream | Kerns et al. 2016 | 0.91 | 0.98 | 0.65 | 0.24 | 0.91 |
| *rs7720298* | stream | Kerns et al. 2016 | 0.46 | 0.24 | 0.15 | 0.73 | 0.91 |
| *rs10497203** | overall toxicity | Fachal et al. 2014 | 0.23 | 0.29 | 0.37 | 0.16 | **0.04** |
| *rs11219068* | overall toxicity | Kerns et al. 2016 | 0.52 | 0.91 | 0.28 | 0.89 | 0.94 |
| *rs12591436* | overall toxicity | Kerns et al. 2016 | 0.40 | 0.60 | 0.19 | 0.25 | 0.66 |
| *rs141799618* | overall toxicity | Kerns et al. 2016 | 0.43 | 0.22 | 0.39 | 0.79 | 0.48 |
| *rs147596965* | overall toxicity | Kerns et al. 2016 | 0.73 | 0.53 | 0.06 | 0.29 | 0.59 |
| *rs264588** | overall toxicity | Fachal et al. 2014 | 0.23 | 0.32 | 0.35 | 0.31 | 0.09 |
| *rs264631** | overall toxicity | Fachal et al. 2014 | 0.35 | 0.38 | 0.31 | 0.37 | 0.08 |
| *rs264651** | overall toxicity | Fachal et al. 2014 | 0.27 | 0.32 | 0.35 | 0.28 | 0.09 |
| *rs2842169* | overall toxicity | Kerns et al. 2016 | 0.22 | 0.81 | **0.03** | 0.72 | 0.45 |
| *rs4775602* | overall toxicity | Kerns et al. 2016 | 0.78 | 0.23 | 0.92 | 0.77 | 0.38 |
| *rs4906759* | overall toxicity | Kerns et al. 2016 | 0.39 | 0.70 | 0.18 | 0.95 | 0.07 |
| *rs6432512** | overall toxicity | Fachal et al. 2014 | 0.21 | 0.31 | 0.36 | 0.19 | 0.08 |
| *rs6535028* | overall toxicity | Kerns et al. 2016 | 0.67 | 0.86 | 0.81 | 0.18 | 0.90 |
| *rs71610881* | overall toxicity | Kerns et al. 2016 | 0.47 | 0.71 | 0.42 | 0.95 | 0.21 |
| *rs7582141** | overall toxicity | Fachal et al. 2014 | 0.21 | 0.31 | 0.36 | 0.19 | 0.08 |
| *rs77530448* | overall toxicity | Kerns et al. 2016 | 0.13 | 0.52 | 0.59 | 0.56 | 0.26 |
| *rs7829759* | overall toxicity | Kerns et al. 2016 | 0.52 | 0.78 | 0.76 | 0.87 | 0.09 |
| *rs79604958* | overall toxicity | Kerns et al. 2016 | 0.37 | 0.66 | 0.54 | 0.23 | 0.24 |
| *rs8075565* | overall toxicity | Kerns et al. 2016 | 0.72 | 0.50 | 0.83 | 0.14 | 0.15 |

* All these variants are highly correlated in European population and represent the same association signal. See also correlation matrix as determined on the Requite population in the Supplementary material, Figure S1.
